# Single-Center Experience with 15 VitalFlow ECMO Deployments for VA- and VV-ECMO Support: Deployment Characteristics, Outcomes, and Complications

**DOI:** 10.3390/jcdd13060233

**Published:** 2026-05-28

**Authors:** Amin Thwairan, Ismail Dalyanoglu, Luis Jaime Vallejo Castano, Esma Yilmaz, Mohammed Morjan, Johanna Wedy, Jamal Azouagh, Mohamed Chiboub, Artur Lichtenberg, Hannan Dalyanoglu

**Affiliations:** 1 Department of Cardiac Surgery, Medical Faculty, Heinrich Heine University, 40225 Dusseldorf, Germany; amin.thwairan@med.uni-duesseldorf.de (A.T.); esma.yilmaz@med.uni-duesseldorf.de (E.Y.); mohammed.morjan@med.uni-duesseldorf.de (M.M.); johanna.wedy@med.uni-duesseldorf.de (J.W.); jamal.azouagh@med.uni-duesseldorf.de (J.A.); mohamed.chiboub@med.uni-duesseldorf.de (M.C.); artur.lichtenberg@med.uni-duesseldorf.de (A.L.); hannan.dalyanoglu@med.uni-duesseldorf.de (H.D.); 2 Sankt Marien Hospital Gelsenkirchen-Buer, 45894 Gelsenkirchen, Germany; i.dalyanoglu@st-augustinus.eu; 3 Cardiovascular Research Institute Düsseldorf (CARID), Medical Faculty, Heinrich Heine University, 40225 Düsseldorf, Germany

**Keywords:** extracorporeal membrane oxygenation, transportable ECMO, Vitalflow, cardiogenic shock, cardiac arrest, VA-ECMO, VV-ECMO

## Abstract

**Background**: Refractory cardiac arrest, cardiogenic shock, and severe acute respiratory failure remain associated with substantial mortality despite advances in advanced life support and extracorporeal membrane oxygenation (ECMO). Transportable ECMO platforms may enable rapid deployment, uninterrupted extracorporeal support, and safer in-hospital transport, but early real-world experience with newer systems remains limited. **Methods**: We conducted a retrospective single-center observational cohort study including all VitalFlow veno-arterial ECMO (VA-ECMO) and veno-venous ECMO (VV-ECMO) deployments performed between November 2025 and March 2026 at a high-volume tertiary cardiac surgery center. Fifteen cases were analyzed, comprising 12 VA-ECMO and 3 VV-ECMO deployments. Data were extracted from electronic health records, perfusion protocols, and ICU documentation. Outcomes included survival to hospital discharge, 30-day survival, neurological outcomes, and complications. Analyses were descriptive. **Results**: The cohort was exclusively male and clinically unstable at implantation, with high lactate and low pH levels consistent with severe hypoperfusion. Median time-to-flow was 33 min, and median ECMO duration was 8 days. Survival to discharge was 60% overall (66.7% VA-ECMO, 33.3% VV-ECMO), with ECMO weaning success in 86.7% and the primary death cause being multiorgan failure (83.3% of non-survivors). All survivors achieving a favorable neurologic outcome (CPC 1). Thirty-day survival was 73.3%. No major bleeding or stroke occurred. Limb ischemia was observed in 4 patients, with 2 patients requiring fasciotomy, all in the VA-ECMO group. Bronchial infection occurred in 3 patients. Lactate levels improved within the first 24 h, and survivors showed a more pronounced metabolic response. **Conclusions**: In this early single-center experience, VitalFlow ECMO was feasible and associated with rapid flow establishment, survival to discharge of 60% of patients, and good neurologic outcome among survivors. The complication profile was acceptable, with limb ischemia as the main adverse event. These findings support further evaluation of this transportable ECMO platform in larger multicenter cohorts.

## 1. Introduction

Extracorporeal membrane oxygenation (ECMO) has become a standard rescue therapy for refractory cardiogenic shock, extracorporeal cardiopulmonary resuscitation (ECPR), and severe acute respiratory distress syndrome (ARDS). Traditional systems require 15–20 min assembly of multiple components (pump, oxygenator, heat exchanger), creating time-critical delays in deployment [1].

Integrated, transportable platforms address these limitations. The LifeBox system (Sorin) represented a prior advance but faced service discontinuation in 2025 across Europe. The VitalFlow ECMO system (Medtronic) offers a pre-assembled single-console design reducing setup time >50% (median 12 vs. 25 min) while supporting flows to 7 L/min with enhanced transport stability [2]. Following CE-mark approval (Q4 2025), high-volume centers transitioned to meet regulatory and clinical deployment requirements. Transportable ECMO platforms are increasingly used across different indications, but the clinical relevance of early real-world data depends on whether outcomes are interpreted within comparable patient groups and modality-specific benchmarks.

Despite technical promise, real-world implementation data from European centers remain limited to manufacturer reports. Routine adoption requires evidence beyond bench testing: deployment feasibility under emergency conditions, complication profiles across VA-/VV-ECMO indications, and early weaning success in rescue populations. This evidence gap is critical as transportable systems move from elective to time-critical applications. The VitalFlow ECMO system is one such device, designed for streamlined extracorporeal support in acute care environments [2]. However, its real-world performance in routine clinical use remains insufficiently described, especially in European centers and across both VA- and VV-ECMO indications. This is relevant because technical feasibility alone does not establish clinical value; deployment characteristics, complication patterns, and early outcomes must also be understood before broader adoption can be justified. We therefore aimed to describe our initial single-center experience (as a high-volume tertiary center with over 200 ECMO cases/year) with VitalFlow ECMO, including an all-comers analysis of initial VitalFlow ECMO deployments, representing the first European implementation focusing on deployment characteristics, management protocols, indications, outcomes, and complications.

## 2. Materials and Methods

### 2.1. Study Design and Setting

This retrospective observational cohort study included all VitalFlow ECMO deployments performed at a high-volume tertiary cardiac surgery center between November 2025 and April 2026. Our center performs more than 200 ECMO cases per year and was among the first European institutions to use this platform in routine practice. This study adhered to the STROBE guidelines for observational studies [3].

### 2.2. Patients and ECMO Configurations

A total of 15 ECMO runs were included: 12 VA-ECMO and 3 VV-ECMO cases. The cohort was analyzed descriptively overall and by ECMO type. No inferential statistics were planned because of the small sample size, particularly in the VV-ECMO subgroup.

### 2.3. Ethics

The study was approved by the local ethics committee (Heinrich Heine University Düsseldorf, Protocol #2023-2527, approved on 9 February 2026) and conducted in accordance with the Declaration of Helsinki. Due to the retrospective and anonymized design, patient consent was waived.

### 2.4. Data Collection

Demographic variables, comorbidities, arrest-related variables, cannulation details, site of implantation, ECMO duration, ICU and hospital length of stay, lactate kinetics, pH values, and complications were collected from electronic records, perfusion documentation, and ICU notes. Model for End-Stage Liver Disease (MELD) scores were calculated at ECMO initiation using serum creatinine, bilirubin, INR, and sodium, and the MELD-I, MELD-II, and MELD-XI variants were recorded as part of the baseline assessment. Key baseline and outcome variables are summarized in Table 1.

### 2.5. Anticoagulation and Monitoring Protocol

Immediately after cannulation, all patients received a 50–80 IU/kg intravenous bolus of unfractionated heparin (UFH) prior to initiating ECMO flow, aiming to achieve a target activated partial thromboplastin time (aPTT) of 50–70 s within the first 4 h. Continuous UFH infusion was subsequently adjusted according to the institutional anticoagulation protocol used during the study period. Monitoring included daily measurements of activated partial thromboplastin time (aPTT), platelet count, fibrinogen, and D-dimer. Anticoagulation was paused in cases of active bleeding (≥2 units PRBC within 6 h) or prior to invasive procedures, with resumption once hemostasis had been achieved. Circuit exchange was performed in the presence of oxygenator thrombosis (pressure gradient >50 mmHg) or persistent clot formation. Suspected heparin-induced thrombocytopenia (HIT) was managed according to institutional protocol with substitution by argatroban [4,5].

### 2.6. Cannulation Protocol

Peripheral VA-ECMO: 25Fr venous/19Fr arterial femoral cannulae (fem-fem 100%) using the Seldinger technique (ultrasound-guided). Distal perfusion catheters (7Fr) applied prophylactically in all arterial cases (100%). Echocardiography confirmed cannula position (tip 2–3 cm IVC/SVC, arterial avoiding aortic root). VV-ECMO: Femoro-jugular (100%) with 23Fr femoral drainage, 19Fr jugular return. Flow management: Target 3.5–4.5 L/min/m^2^.

### 2.7. Weaning from ECMO and Complication Surveillance

Weaning criteria were defined as follows: 24 h stability at <2.0 L/min with lactate <2 mmol/L, ScvO2 >70%, cardiac power output >0.8 W. Decannulation: Surgical (OR, 75%), percutaneous bedside (25%). Complication surveillance: Daily aPTT/platelets/fibrinogen/LDH, distal perfusion Doppler (ABI > 0.8), circuit pressures (ΔP < 50 mmHg oxygenator).

### 2.8. Definition of Outcomes

Primary outcomes were survival to hospital discharge and favorable neurologic outcome, defined as Cerebral Performance Category (CPC) 1–2. Secondary outcomes included 30-day survival, ECMO duration, ICU length of stay, hospital length of stay, lactate clearance, pH change, and major complications.

### 2.9. Statistical Analysis

All variables are presented descriptively. Continuous data are reported as median with interquartile range and overall range; categorical variables as counts and percentages. No hypothesis testing was performed.

## 3. Results

The cohort was reported as an overall early VitalFlow experience; however, given the mechanistic and prognostic differences between VA-ECMO and VV-ECMO, VA-ECMO represents the clinically most informative subgroup, whereas the VV-ECMO cases are too few for meaningful performance benchmarking.

### 3.1. Baseline Characteristics

The cohort consisted of 15 patients, all male, with a median age of 53 years. This exclusively male composition likely reflects the small sample size and the local case mix during the study period rather than a predefined eligibility restriction. Most patients were critically ill at the time of ECMO implantation, with a median lactate of 13.6 mmol/L and median pH of 7.1. Median time to flow was 33 min overall, indicating a relatively rapid deployment process. The majority of cannulations were femoro-femoral, and most implantations occurred in the ICU (Table 1).

### 3.2. Detailed Indications

VA-ECMO indications comprised ECPR (*n* = 3, 25%) initiated in the emergency department and refractory cardiogenic shock (*n* = 9, 75%) in the ICU. Transthoracic echocardiography at initiation showed LVEF 18% (IQR 15–22%) and severe RV dysfunction (TAPSE < 12 mm) in 8/12 (67%). VV-ECMO patients met institutional ARDS criteria (PaO2/FiO2 < 80, Murray score 3–4), representing severe pneumonia (*n* = 2) and post-traumatic ARDS (*n* = 1) (Table 2).

### 3.3. Outcomes and Complications

Overall survival to discharge was 60% (9/15), and all survivors had a favourable neurological outcome (CPC 1). Survival to discharge was 66.7% in VA-ECMO (8/12) and 33.3% in VV-ECMO (1/3), but the VV-ECMO subgroup is too small for meaningful comparative interpretation. Successful weaning from ECMO was achieved in 13/15 patients (86.7% overall; 91.7% VA-ECMO, 66.7% VV-ECMO). All patients (100%) were managed with a bridge-to-recovery strategy (Table 2). Median ECMO duration was 8 days, median ICU stay was 12 days, and median hospital stay was 51 days. Among the 6 non-survivors, time from admission to death was 40 days (IQR 26.5; range 8–105 days) and from explantation to death was 8.5 days (IQR 1; range 0–47 days). Primary causes of death were multiorgan failure (5/6, 83.3%) and neurological injury (1/6, 16.7%) (Table 3). No major bleeding or stroke occurred. Limb ischemia occurred in 4 patients (26.7%), with 2 requiring fasciotomy, all within the VA-ECMO subgroup, and bronchial infection occurred in 3 patients (Table 3). Limb ischemia was managed with distal perfusion catheters in all 4 affected cases, and 2 patients required fasciotomy. No oxygenator exchanges were required.

All patients received an immediate heparin bolus (median 65 IU/kg) upon cannulation completion, achieving the target aPTT (50–70 s) within 4 h without excess anticoagulation. Anticoagulation pauses occurred in 4 patients (26.7%) for minor bleeding, but no circuit exchanges or heparin-induced thrombocytopenia were observed (Table 4).

**Figure 1:**
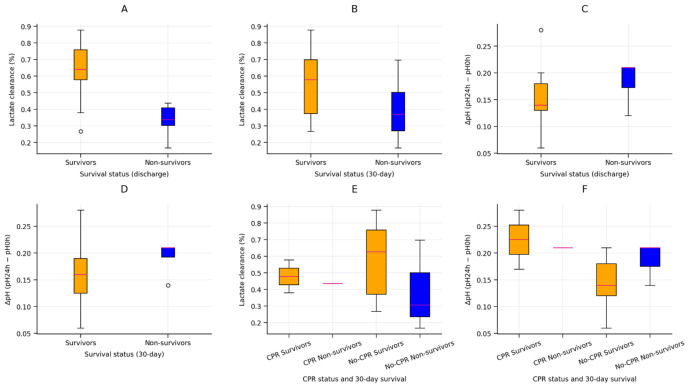
Lactate clearance and pH change according to survival and CPR status. Panels (**A**,**B**) show lactate clearance (%) stratified by survival to hospital discharge and 30-day survival, respectively. Panels (**C**,**D**) show ΔpH (pH24 h − pH0 h) according to the same survival endpoints. Panels (**E**,**F**) present sensitivity analyses stratified by cardiopulmonary resuscitation (CPR) prior to ECMO initiation and 30-day survival. Survivors are shown in orange and non-survivors in blue. Boxplots display median values with interquartile ranges.

Figure 1 summarizes lactate kinetics and acid–base changes within the first 24 h after ECMO implantation. Lactate clearance was significantly higher in survivors compared with non-survivors both at hospital discharge and at 30 days (Panels A and B). Similarly, survivors demonstrated a greater normalization of arterial pH, reflected by higher ΔpH values at both survival endpoints (Panels C and D). In sensitivity analyses stratified by cardiopulmonary resuscitation (CPR) status, these associations remained consistent: among patients with and without prior CPR, survivors exhibited greater lactate clearance and a superior pH recovery compared with non-survivors (Panels E and F).

### 3.4. Metabolic Response

The metabolic response after ECMO initiation was favorable overall. Lactate decreased from a median of 13.6 mmol/L at implantation to 6.4 mmol/L at 24 h, and median pH improved from 7.1 to 7.3. Survivors showed a stronger lactate decline and greater lactate clearance than non-survivors, suggesting that early biochemical response may reflect the adequacy of circulatory support.

## 4. Discussion

The present cohort combines VA-ECMO and a very small VV-ECMO subgroup, and because these modalities differ substantially in indication, severity profile, and expected outcomes, the VV-ECMO cases should be interpreted as descriptive rather than benchmark data.

This early single-center study suggests that VitalFlow ECMO can be deployed rapidly and with a clinically acceptable safety profile in both VA- and VV-ECMO settings. The cohort was severely ill, with substantial metabolic derangement at implantation, yet overall survival to discharge was 60%, and all survivors had favourable neurological recovery. Despite 20% of patients having CCI ≥ 5, all with pre-existing multimorbidity’s survived, underscoring ECMO effectiveness [6,7]. These results are clinically notable because the cohort was composed largely of critically unstable patients requiring urgent extracorporeal support.

The median time-to-flow of 33 min indicates efficient on-site implementation. In ECMO-dependent rescue scenarios, shortening the interval between arrest or shock and effective flow is crucial, as earlier perfusion restoration is likely associated with better organ recovery. The observed lactate reduction and pH normalization support the physiologic effectiveness of the circuit. Beyond demonstrating technical feasibility, the early metabolic response observed in this cohort provides important prognostic insight. As summarized in Figure 1, survivors showed significantly higher lactate clearance and more pronounced pH normalization within the first 24 h after ECMO implantation, both at hospital discharge and at 30 days. Notably, these associations persisted in sensitivity analyses accounting for cardiopulmonary resuscitation prior to ECMO initiation, indicating that dynamic metabolic recovery conveys prognostic information even in patients with profound circulatory collapse.

The 86.7% ECMO weaning success (91.7% VA-ECMO) and 100% bridge-to-recovery strategy demonstrate effective circulatory support in this high-acuity cohort. Multiorgan failure predominated among non-survivors (83.3%), consistent with ECMO rescue failure patterns where persistent organ dysfunction exceed device capabilities [6]. The median 8.5-day explanation-to-death interval confirms adequate bridge periods prior to terminal decline.

Lactate clearance >50% within 24 h was observed more frequently among survivors and may be indicative of a favorable outcome, in line with prior prognostic studies [8]. A numerically higher lactate clearance was also noted in survivors. The potential prognostic relevance of early lactate kinetics observed in this cohort appears consistent with—and may extend—prior ECMO and ECPR literature.

Several studies have suggested that lactate clearance within the first 24 h after ECMO initiation may be a more informative marker of short- and mid-term survival than single admission lactate values [9,10]. More recently, a large multicenter analysis of ECPR patients reported that 24 h lactate clearance was independently associated with one-year survival after adjustment for arrest characteristics and comorbidity burden, highlighting its potential robustness across high-risk populations [11].

Our findings are in general agreement with these observations, despite the smaller cohort size, and show a similar pattern in patients with antecedent CPR. Together, these findings may suggest that early dynamic metabolic changes reflect the effectiveness of systemic perfusion recovery rather than baseline illness severity alone.

The immediate heparin bolus strategy post-cannulation was safely implemented even in post-CPR patients (*n* = 3), with no major bleeding despite profound initial hypoperfusion (median lactate 13.6 mmol/L). This contrasts with historical concerns about early anticoagulation in coagulopathic arrest patients, where delayed strategies have been advocated [4]. Our aPTT target of 50–70 s achieved therapeutic levels rapidly (median time to target 4 h) without excess anticoagulation, as evidenced by zero major bleeding events. The 26.7% anticoagulation pause rate reflects responsive management of minor bleeding sources, with timely resumption preventing circuit thrombosis (no exchanges required) [4,12].

The absence of major bleeding and stroke is reassuring, although limb ischemia in the VA-ECMO group highlights the persistent vascular trade-off of peripheral arterial cannulation. The rate observed here is in the expected range for VA-ECMO complications described in the literature [13].

Limb ischemia management included distal perfusion catheters in all 4 affected cases, with 2 requiring fasciotomy. This 33.3% rate in VA-ECMO aligns with peripheral cannulation literature and was not associated with worsened survival [5]. The absence of HIT and circuit thrombosis reflects effective monitoring and UFH management in this high-risk cohort [5,14,15].

Bronchial infection and one case of marked hemolysis were additional complications, but no oxygenator exchanges were necessary. Zero oxygenator exchanges despite an 8-day median duration suggest excellent circuit biocompatibility, consistent with VitalFlow’s low-thrombogenicity design [2]. This is particularly relevant for transport scenarios where circuit failure can jeopardize patient safety [16].

MELD scores [median MELD-I 18 (14–24)] indicated moderate liver dysfunction at baseline, yet rapid lactate clearance suggests preserved hepatic reserve under ECMO support. MELD-II > 25 identified high-risk patients, all of whom survived, supporting early ECMO initiation before irreversible liver injury [17]. The median CCI of 2.0 reflects the low-moderate comorbidity burden typical of ECMO rescue populations primarily presenting with acute organ failure [6]. Nevertheless, all 3 patients with CCI ≥ 5 (75th percentile) survived, demonstrating ECMO effectiveness even in pre-morbid patients. This aligns with ELSO registry data where CCI > 4 doubles mortality risk [6].

Our findings are broadly consistent with contemporary ECMO transport and deployment literature showing that mobile ECMO systems can be used safely in high-acuity settings when managed by experienced teams. As this is an early report from a single center, the results should be interpreted cautiously. Larger multicenter studies are needed to determine whether the apparent feasibility and favourable neurological outcomes are reproducible across different settings and cases. These findings align with mobile ECMO programs emphasizing rapid deployment [16,18].

## 5. Limitations

This study has several limitations. Firstly, the small sample size, especially in the VV-ECMO subgroup, which precludes formal statistical comparison. Secondly, the retrospective design and the fact that it is a single-center study, which limits generalizability. Third, the exclusively male cohort limits generalizability to female patients and broader ECMO populations. Fourth, the user-reported handling characteristics were not based on a validated questionnaire. Fifth, historical comparison with the prior Lifebox system omitted due to systematic differences in device generation, team experience, and patient selection evolution during 5-year interval. Finally, because the analysis reflects early implementation, outcomes may partly reflect local team expertise rather than device performance alone.

## 6. Conclusions

In this initial single-center experience, VitalFlow ECMO deployment was feasible across VA- and VV-ECMO indications and was associated with rapid flow establishment, favourable survival, and good neurological outcome among survivors. The complication profile was acceptable, with limb ischemia as the main adverse event. These findings support further evaluation in larger multicenter cohorts.

## Figures and Tables

**Table 1:** Baseline Characteristics.

Variable	Overall (*n* = 15)	VA-ECMO (*n* = 12)	VV-ECMO (*n* = 3)
**Age, years**	53.0 (42.5–64.5; 18–80)	55.5 (51.8–63.8; 28–70)	28.0 (23.0–54.0; 18–80)
**Male sex**	15 (100%)	12 (100%)	3 (100%)
**BMI ^1^** **, kg/m^2^**	28.3 (26.7–31.7; 20.4–35.5)	29.4 (27.6–32.4; 25.9–35.5)	21.9 (21.2–25.0; 20.4–28.1)
**BSA ^2^** **, m^2^**	2.1 (2.0–2.3; 1.5–2.4)	2.2 (2.1–2.3; 1.9–2.4)	1.9 (1.7–1.9; 1.5–2.0)
**Charlson Comorbidity Index** **(CCI) ^3^**	2.0 (2.0–5.5; 2–8)	3.0 (2.0–6.2; 2–8)	2.0 (2.0–3.0; 2–4)
**Shock**	7 (46.7%)	5 (41.7%)	2 (66.7%)
**CPR ^4^**	3 (20%)	3 (25%)	0 (0%)
**CPR duration, min**	40.0 (35.0–45.0; 30–50)	40.0 (35.0–45.0; 30–50)	—
**Initial rhythm**			
**Sinus**	12 (80%)	9 (80%)	3 (100%)
**AF ^5^**	3 (20%)	3 (20%)	0 (0%)
**non-shockable**	15 (100%)	12 (100%)	3 (100%)
**No-flow time, min**	0.0 (0.0–0.0; 0–10)	0.0 (0.0–2.5; 0–15)	0.0 (0.0–0.0; 0–0)
**Low-flow time, min**	35.0 (32.5–37.5; 30–40)	35.0 (32.5–37.5; 30–40)	—
**Call-to-ECMO, min**	10.0 (8.5–11.0; 8–12)	10.0 (8.0–11.0; 8–12)	9.0 (9.0–11.5; 9–14)
**Time-to-flow, min**	33.0 (25.5–33.5; 19–36)	33.0 (27.2–33.2; 20–36)	33.0 (26.0–35.0; 19–37)
**Lactate at implantation, mmol/L**	13.6 (10.2–18.6; 5.9–23.5)	12.2 (9.9–16.7; 5.9–23.5)	20.0 (16.6–20.0; 13.2–20.0)
**pH ^6^ at implantation**	7.1 (7.0–7.2; 6.92–7.32)	7.2 (7.0–7.2; 6.9–7.3)	7.1 (7.1–7.1; 7.0–7.2)
**Ventilation before ECMO, days**	1.0 (0.5–2.0; 0–6)	1.0 (0.8–3.2; 0–6)	2.0 (1.0–3.0; 0–4)
**Cannulation site fem-fem/fem-jug**	12 (80%)/3 (20%)	12 (100%)/0 (0%)	0 (0%)/3 (100%)
**Implant location ICU ^7^** **/ER ^8^**	12 (80%)/3 (20%)	9 (75%)/3 (25%)	3 (100%)/0 (0%)
**MELD-I ^9^**	18 (14–24; 8–35)	19 (15–25; 10–35)	15 (12–18; 8–22)
**MELD-II ^10^**	22 (17–28; 11–39)	23 (18–29; 13–39)	18 (14–22; 11–25)
**MELD-XI ^11^**	16 (12–21; 7–37)	17 (13–22; 9–31)	13 (10–17; 7–20)

^1^ BMI = body mass index, ^2^ BSA = body surface area, ^3^ CCI = Charlson Comorbidity Index, ^4^ CPR = cardiopulmonary resuscitation, ^5^ AF = atrial fibrillation, ^6^ ph = pH, ^7^ ICU = intensive care unit, ^8^ ER = emergency room, ^9^ MELD-I = Model for End-Stage Liver Disease and uses creatinine/bilirubin/INR, ^10^ MELD-II includes Na, ^11^ MELD-XI excludes INR. Baseline MELD scores indicated moderate liver dysfunction [MELD-I: 18 (14–24; 8–35), MELD-II: 22 (17–28; 11–39), MELD-XI: 16 (12–21; 7–31)], while the Charlson Comorbidity Index [CCI: 2.0 (2.0–5.5; 2–8)] reflected low-moderate comorbidity burden with 20% (*n* = 3) CCI ≥ 5.

**Table 2:** Detailed ECMO indications by subgroup.

Variable	Overall (*n* = 15)	VA ^1^-ECMO (*n* = 12)	VV ^2^-ECMO (*n* = 3)
**eCPR ^3^ (Emergency Department)**	3 (20.0%)	3 (25.0%)	0 (0%)
**Refractory cardiogenic shock (ICU ^4^** **)**	9 (60.0%)	9 (75.0%)	0 (0%)
**Severe ARDS ^5^ (P/F ^6^ < 80)**	3 (20.0%)	0 (0%)	3 (100.0%)
**LVEF ^7^** **. % at initiation**		18% (15–22%)	
**Severe right ventricular failure**		8/12 (67.0%)	
**VV ^2^** **-ECMO** **threshold**			Murray **^8^** 3–4

^1^ VA-ECMO = veno-arterial extracorporeal membrane oxygenation, ^2^ VV-ECMO = veno-venous extracorporeal membrane oxygenation, ^3^ eCPR = extracorporeal cardiopulmonary resuscitation, ^4^ ICU = Intensive Care Unit, ^5^ ARDS = adult respiratory distress syndrom, ^6^ P/F = ratio of partial pressure of oxygen to fraction of inspired oxygen, ^7^ LVEF = Left Ventricular Ejection Fraction, ^8^ Murray = lung injury score.

**Table 3:** Outcomes and complications.

Variable	Overall (*n* = 15)	VA-ECMO (*n* = 12)	VV-ECMO (*n* = 3)
**ECMO duration, days**	8.0 (4.5–10.5; 1–21)	7.0 (3.8–11.2; 1–21)	10.0 (7.5–10.0; 5–10)
**ICU ^1^ length of stay, days**	12.0 (10.5–22.5; 1–36)	13.5 (10.8–22.2; 1–36)	11.0 (9.5–17.5; 8–24)
**Hospital length of stay, days**	51.0 (27.5–66.5; 8–105)	54.5 (35.0–65.8; 8–91)	20.0 (18.0–62.5; 16–105)
**Survival to discharge**	9 (60%)	8 (66.7%)	1 (33.3%)
**30-day survival**	11 (73.3%)	9 (75%)	2 (66.7%)
**Bridge strategies**			
**Bridge to recovery**	15/15 (100.0%)	12/12 (100.0%)	3/3 (100.0%)
**Died on ECLS/ECMO**	2 (13.3%)	1 (8.3%)	1 (33.3%)
**Died after decannulation before discharge**	4 (26.6%)	3 (25.0%)	1 (33.3%)
**Primary cause of death**			
**Multiorgan failure**	5 (83.3%)	3 (75.0%)	2 (100.0%)
**Neurological injury**	1 (16.6%)	1 (25.0%)	0 (0%)
**Death timing, days (median, range)**			
**Admission to death**	40 (26.5; 8–105)	29.75 (26.5; 8–47)	60.5 (61; 16–105)
**Explantation to death**	8.5 (1; 0–47)	12.25 (1: 0–47)	1 (1; 0–2)
**Favorable neurological outcome (CPC ^2^ 1–2)**	9 (60%)	8 (66.7%)	1 (33.3%)
**Lactate at 24 h, mmol/L**	6.4 (3.4–13.1; 1.7–14.8)	5.5 (3.0–10.4; 1.7–14.8)	12.6 (8.3–13.2; 4.0–13.9)
**Lactate clearance, %**	44 (34–67; 17–88)	51 (36–67; 17–88)	37 (34–53; 30–70)
**Delta Lactate, mmol/L**	6.4 (5.0–8.5; 2.4–12.1)	6.4 (3.9–8.3; 2.4–12.1)	7.4 (6.7–8.3; 6.1–9.2)
**pH at 24 h**	7.3 (7.2–7.3; 7.1–7.4)	7.3 (7.2–7.3; 7.1–7.4)	7.3 (7.2–7.3; 7.2–7.3)
**Delta pH**	0.2 (0.1–0.2; 0.1–0.3)	0.2 (0.1–0.2; 0.1–0.3)	0.1 (0.1–0.2; 0.1–0.2)
**Major bleeding**	0 (0%)	0 (0%)	0 (0%)
**Stroke**	0 (0%)	0 (0%)	0 (0%)
**Limb ischemia**	4 (26.7%)	4 (33.3%)	0 (0%)
**Requiring fasciotomy**	2 (13.3%)	2 (50.0%)	
**Bronchial infection**	3 (20%)	3 (25%)	0 (0%)
**Hemolysis (LDH ^3^)**	323 (251.5–468; 16–9776)	330.5 (271.2–559.2; 157–9776)	323 (169.5–378.5; 16–434)

^1^ ICU = intensive care unit, ^2^ CPC = Cerebral Performance Category, ^3^ LDH = Lactatdehydrogenase.

**Table 4:** Anticoagulation Parameters and Management.

Parameter	Overall (*n* = 15)	VA-ECMO (*n* = 12)	VV-ECMO (*n* = 3)
**Heparin bolus at cannulation**	15 (100%)	12 (100%)	3 (100%)
**Initial bolus dose, IU/kg**	65 (55–75; 50–80)	67 (58–78; 55–80)	60 (52–65; 50–65)
**Target aPTT ^1^** **, seconds**	50–70	50–70	50–70
**Median aPTT day 1, seconds**	58 (52–65; 45–72)	59 (54–66; 48–72)	55 (50–60; 45–62)
**Anticoagulation hold**	4 (26.7%)	3 (25%)	1 (33.3%)
**Circuit exchanges**	0 (0%)	0 (0%)	0 (0%)
**Suspected HIT ^2^**	0 (0%)	0 (0%)	0 (0%)

^1^ aPTT = activated partial thromboplastin time, ^2^ HIT = heparin-induced thrombocytopenia.

## Data Availability

The data underlying this study were obtained retrospectively from clinical records and contain sensitive personal health information. Due to privacy regulations and ethical considerations, the raw data cannot be made publicly available. Researchers with a legitimate interest may request access to anonymized or aggregated data, subject to approval by the responsible ethics committee and in compliance with applicable data protection laws.
